# Relationship between Tumor Budding and Partial Epithelial–Mesenchymal Transition in Head and Neck Cancer

**DOI:** 10.3390/cancers15041111

**Published:** 2023-02-09

**Authors:** Kohei Okuyama, Keiji Suzuki, Souichi Yanamoto

**Affiliations:** 1 Department of Periodontics and Oral Medicine, School of Dentistry, University of Michigan, 1011 North University Ave, Ann Arbor, MI 48109, USA; 2 University of Michigan Rogel Cancer Center, 1600 Huron Pathway, Ann Arbor, MI 48105, USA; 3 Department of Oral and Maxillofacial Surgical Oncology, Graduate School of Medical and Dental Sciences, Tokyo Medical and Dental University, 1-5-45, Yushima, Bunkyo-ku, Tokyo 113-8510, Japan; 4 Department of Radiation Medical Sciences, Atomic Bomb Disease Institute, Nagasaki University, 1-12-4, Sakamoto, Nagasaki 852-8523, Japan; 5 Department of Oral Oncology, Graduate School of Biomedical and Health Sciences, Hiroshima University, 1-2-3, Kasumi, Minami-ku, Hiroshima 734-8553, Japan

**Keywords:** head and neck squamous cell carcinoma, tumor budding, partial epithelial–mesenchymal transition, tumor microenvironment, cancer-associated fibroblast

## Abstract

**Simple Summary:**

In this article, we outline updates on the current relevance of tumor budding (TB) and partial epithelial–mesenchymal transition (p-EMT), both as a prognostic marker in the tumor microenvironment (TME) of head and neck squamous cell carcinoma (HNSCC). Although these individual pieces of evidence have been well investigated, no report has focused on their organized functional relationships. Understanding the mechanism of TB onset and the relationship between p-EMTs may facilitate the development of novel diagnostic and prognostic methods, and targeted therapies for the prevention of invasion and metastasis.

**Abstract:**

Tumor budding (TB), a microscopic finding in the stroma ahead of the invasive fronts of tumors, has been well investigated and reported as a prognostic marker in head and neck squamous cell carcinoma (HNSCC). Epithelial–mesenchymal transition (EMT) is a crucial step in tumor progression and metastasis, and its status cannot be distinguished from TB. The current understanding of partial EMT (p-EMT), the so-called halfway step of EMT, focuses on the tumor microenvironment (TME). Although this evidence has been investigated, the clinicopathological and biological relationship between TB and p-EMT remains debatable. At the invasion front, previous research suggested that cancer-associated fibroblasts (CAFs) are important for tumor progression, metastasis, p-EMT, and TB formation in the TME. Although there is biological evidence of TB drivers, no report has focused on their organized functional relationships. Understanding the mechanism of TB onset and the relationship between p-EMTs may facilitate the development of novel diagnostic and prognostic methods, and targeted therapies for the prevention of metastasis in epithelial cancer. Thus far, major pieces of evidence have been established from colorectal cancer (CRC), due to a large number of patients with the disease. Herein, we review the current understanding of p-EMT and TME dynamics and discuss the relationship between TB development and p-EMT, focusing on CAFs, hypoxia, tumor-associated macrophages, laminin–integrin crosstalk, membrane stiffness, enzymes, and viral infections in cancers, and clarify the gap of evidence between HNSCC and CRC.

## 1. Introduction

Tumor budding (TB), a well-investigated microscopic finding existing in the stroma ahead of the invasive front of a tumor, is a prognostic marker for various types of epithelial cancers. TB is defined as the presence of isolated small clusters of cancer cells, including up to five cells [1]. From the previous evidence, high-intensity TB represents a more aggressive and increasingly malignant potential of head and neck squamous cell carcinoma (HNSCC), increased cervical lymph node metastases (LNM) [2,3,4], and poor survival (overall, disease-specific, and disease-free survival rates), especially in patients with early-stage oral SCC [5,6]. In addition, two meta-analyses confirmed the importance of TB in the clinical outcomes of oral SCC [2,7]. This has also been demonstrated in other solid tumors, including colorectal cancer (CRC) [8,9,10], esophageal SCC [11,12], gastrointestinal carcinoma [13], breast cancer (BC) [14], lung SCC and adenocarcinoma [15,16], intrahepatic cholangiocarcinoma [17,18], gastric [19], cervical and endometrial cancer [20,21,22], bladder [23], and melanoma [24].

TB formation has been proposed as evidence of epithelial–mesenchymal transition (EMT), which is widely recognized as related to cancer cell migration and a step required for metastasis [25]. Moreover, TB cells possess cellular plasticity properties such as cytoskeletal deformability and motility [26]. Immunohistochemical (IHC) staining showed that TB is associated with reduced E-cadherin and increased vimentin expression [27]. In addition, molecular analyses have provided details regarding the genetic background of TB [28,29,30,31], and genetic analyses of HNSCC have furthered our understanding of this phenomenon [32,33]. 

Recently, a “partial” epithelial–mesenchymal transition (p-EMT), which is a hybrid state, was reported wherein both epithelial and mesenchymal characteristics can be observed simultaneously [18]. IHC quantification of p-EMT in HNSCC primary tumors is also reliably associated with aggressive tumor states, including LNM, perineural invasion, and high-grade tumors. The identification of p-EMT biomarkers may aid in decision-making for adjuvant therapy planning and/or performing elective neck dissection in the N0 neck [34,35]. p-EMT can also lead to the development of circulating tumor cells and drug resistance [36,37]. 

EMT is a crucial step in tumor progression and metastasis and cannot be separated from the relationship between TB and p-EMT. In this review, we summarize the current understanding of TB and p-EMTs and discuss their relationship and contributions to the tumor microenvironment (TME).

## 2. EMT Status and Stemness on TB in HNSCC

The ability of TB cells to migrate has been suggested based on the strong association between high-grade TB and metastasis in various types of cancers, including different subtypes of HNSCC [2,38,39]. By comparing neoplastic cells in the tumor mass and those micro-dissected from TB cells, De Smedt et al. found 296 differentially expressed genes, revealing that TB cells undergo phenotype switching while detaching from the main tumor, as well as the upregulation of genes related to cell motility and downregulation of genes involved in cell growth and proliferation [40]. Moreover, in nasopharyngeal carcinoma, TB cells show high expression of the cancer stem cell (CSC) marker aldehyde dehydrogenase 1 (ALDH1), a cytosolic enzyme that promotes the intracellular oxidation of aldehydes and contributes to the oxidation of retinol to retinoic acid in the initial stages of stem cell differentiation [41]. Retinoic acid is directly implicated in the management of cell proliferation [42]. It has been proposed that such TB cells might have invasive and metastatic characteristics and may be a prognosticator for poor survival [43]. Moreover, Marangon Junior et al. found that in tumors with high-grade TB cells, the expression of ALDH1 was higher in the TB area than it was outside the TB region, suggesting that this pattern of ALDH1 expression is a consequence of cancer cells undergoing TB with the phenotypic properties of CSCs [44]. 

TB in HNSCC has also shown a strong correlation with the overexpression of CD44, including its variants, which are known to play a role in the regulation of cell proliferation and migration [4,45]. TB has been reported to correlate strongly with EMT in HNSCC; cells undergoing EMT have been identified in TB regions [46]. In addition to a decrease in E-cadherin, vimentin, *ZEB1*, and *PRRX1*, all of which induce EMTs, were higher in TB cells than in the main tumor mass [47,48]. Furthermore, RNA sequencing analysis of HNSCC revealed that cells in the TB regions express factors involved in transforming growth factor-β (TGF-β) signaling, indicating that TB represents a shift toward the EMT phenotype [47]. Histologically, rounded and spindle-like cells are typically more common in TB regions [48]. 

On the other hand, Sharaf et al. reported that ALDH1A1 and CD44 were expressed to a comparable extent in healthy mucosa and cancerous tissues [49]. Moreover, Jacob et al. reported that CD44 indicated a significantly worse prognosis for survival, while ALDH1 had protective properties in HNSCC survival [50], indicating that they are not prognostic in all HNSCC cohorts. 

Moreover, Wang et al. reported that high-density cytocapsular tubes (CTs), which provide membranous channels for HNSCC cell interconnection and multidirectional locomotion, were significantly associated with T stage, LNM, differentiation, depth of invasion, TB development, TNM stage, and recurrence [51]. In addition, high CT density indicated decreased overall survival and progression-free survival in patients with HNSCC. They found that CTs were present in human primary HNSCC tissues with various patterns, morphologies, and locations but were not detected in the normal oral epithelium. 

Taken together, TB cells have been shown to be involved in EMT and CSC formation. 

## 3. Evidence of p-EMT

**Figure 1:**
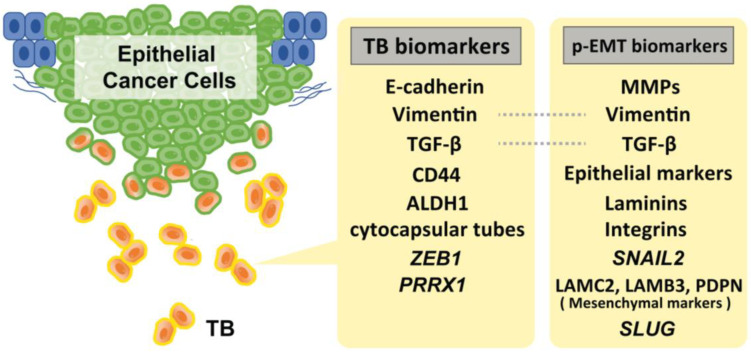
Representative biomarkers of TB and p-EMT. Of note, *SNAIL2* amplification is strongly specific to the p-EMT status. Although several markers for these statuses have been reported, there is a lack of verification of the specificity of these markers to distinguish TB and p-EMT status.

During TB cluster advancement, intercellular adherence junctions and cadherins mediate multicellular integrity and cell–cell coordination. Extracellular matrix metalloproteinases (MMPs) and basement membrane type IV collagens are essential for tracking, clearing, and remodeling the secondary extracellular matrix [2]. The cells in the p-EMT express a mixture of epithelial and mesenchymal features. Recent studies suggested that *SNAIL2* is the first transcription factor upregulated in EMT, suggesting that this program is a p-EMT, rather than a full EMT [52,53]. Moreover, recent single-cell transcriptome analyses have identified several genes that are involved in p-EMT. Puram et al. found that >70% of oral cancers in The Cancer Genome Atlas (TCGA) are of the malignant–basal type, which displays either an EMT or p-EMT as a hallmark. They also investigated a subset of cells that also expressed a p-EMT program with extracellular matrix proteins but lacked classical EMT transcription factors. While the program had key features of classical EMT, it lacked other hallmarks: (i) the overall expression of epithelial markers was maintained, (ii) the expression of the classical EMT transcription factors, *ZEB1/2*, *TWIST1/2*, and *SNAIL1* were not detected, and (iii) only *SNAIL2* was detected in their cohort. However, while its expression correlated with the program across tumors, it did not correlate with the program across individual cells within a tumor. Furthermore, they also found that p-EMT cells were localized to the leading edge of primary tumors in proximity to cancer-associated fibroblasts (CAFs). They added that CAFs abundance does not independently predict LNM; however, tumors with high scores for both CAF and p-EMT have a high propensity for metastasis, consistent with a cooperative effect [53]. Moreover, subsequent experiments using quantitative IHC assays (for PDPN, LAMB3, and LAMC2) further revealed that p-EMT is statistically associated with LNM and perineural invasion in 99 primary oral cancer tissues, suggesting that p-EMT is a useful indicator for decision-making in adjuvant therapy [34]. Other, Schinke et al. reported that SLUG protein levels in HNSCC predicted disease-free survival, and its peripheral expression at the interphase to the TME was significantly increased in relapsing patients. Taking previous evidence into consideration, they suggested SLUG is not only a surrogate for p-EMT, but rather actively contributes to inducing a p-EMT phenotype in HNSCC [54]. Figure 1 shows the representative biomarkers of TB and p-EMT.

## 4. Plasticity and EMT

Yang et al. recommended the use of the term “epithelial–mesenchymal plasticity” (EMP) to describe the ability of cells to adopt mixed E/M features and to interconvert intermediate E/M phenotypic states arrayed along an epithelial–mesenchymal spectrum [55]. In our previous study, we identified the concurrent expression of cytokeratin-14 (CK14) in vimentin-positive early-stage tongue SCC cells. Epithelial markers, including E-cadherin, were also expressed in some vimentin-positive cancer cells. Loss of membranous localization of E-cadherin is a hallmark of EMT. However, the concurrent expression of CK14 and vimentin is not defined as EMT or p-EMT [56]. In addition, Kohler et al. reported that vimentin levels in TB cells do not correlate with E-cadherin levels [57], suggesting that the disappearance of E-cadherin does not always indicate EMT. Similar to EMT-defined cells, anaplastic cells also express both epithelial and mesenchymal markers; however, EMT and anaplasticity are entirely different. Notably, we believe that p-EMT includes both CK^+^ and CK^−^ patterns and defined the CK^+^ pattern as an anaplastic transition (APT). We also found that the number of CAFs in the stroma surrounding SCC increased significantly near APT cells, resulting in local recurrence in early-stage tongue SCCs [56]. This finding will inform future treatments targeting the tumor stroma. Pathologically, anaplastic states are usually observed in thyroid cancer; anaplastic thyroid cancer accounts for only 1–2% of all thyroid cancers and 40–50% of all thyroid cancer deaths. Owing to its highly aggressive characteristics (e.g., quick metastasis), anaplastic thyroid cancer is an important clinical challenge [58,59,60]. In CRC, Meyer et al. suggested that the CK^+^/vimentin^+^ stroma may be of mesothelial origin and show features of mesenchymal cells and CAFs. These characteristics of the atypical stroma are associated with metastasis of CRC via mesothelial–mesenchymal transition [61]. This may be the same observation that we proposed as the APT concept. Thus, CAFs play a necessary role in modulating the TME to make metastatic fields deeply related to p-EMT and function as intermediates that drive EMT (details of CAFs are also described later). 

## 5. Immunity Relating to TB Formation

The mechanism of how TB cells evade immune cells during their development is still poorly understood. However, recent evidence has revealed that specific interactions between immune cells in the TME affect the formation of TBs. TBs are inversely correlated with CD8^+^ T-cell infiltration; thus, a T-cell–based defense against TB cells to inhibit their formation in the TME has been basically indicated. Moreover, in pancreatic ductal adenocarcinoma (PDAC), increased TB is associated with increased accumulation of FOXP3^+^ T cells in the TME, suggesting a strong association between CD163^+^ M2 macrophages and TB development [62]. One study focused on the prognostic significance of PDAC and divided patients into three subtypes clinically and biologically: immune-escape, immune-rich, and immune-exhausted. The immune-escape category was characterized by a high mutation rate in *CDKN2A*, *SMAD4*, and *PIK3CA*, high-grade TB, and a TME that is rich in FOXP3^+^ T_regs_ and poor in T and B cells. The study also reported differences between the immune escape and immune-rich subtypes, which seem to have opposite microenvironmental response patterns and mutational backgrounds [63]. In HNSCC, Chen et al. investigated 276 HNSCC samples via next-generation sequencing and reported that the most frequently mutated genes were *TP53* (65%), *PIK3CA* (16.8%), *CDKN2A* (12.8%), *HRAS* (9.3%), *BRAF* (9.0%), *EGFR* (6.7%), and *FGFR3* (5.8%). They also revealed that genetic mutations located in 14 genes (*ABL1*, *AKT1*, *BRAF*, *CTNNB1*, *FGFR3*, *HRAS*, *KIT*, *MPL*, *NOTCH1*, *PTEN*, *PTPN11*, *SMAD4*, *STK11*, and *VHL*) were significantly associated with disease-free survival [64]. According to Moreira A et al., frequent molecular alterations in HNSCC included the *TP53* (71%), *TERT* promoter (50%), *CDKN2A* (25%), *FAT1* (17%), *PIK3CA* (14%), and *NOTCH1* (15%) genes [65]. Although the origin of the tissue is different between HNSCC and PDAC, *CDKN2* is the common high-frequent mutation relating to poor survival in both cancers and may include a key factor for developing TBs. Moreover, Sadozai et al. reported that PDAC cases with high-grade TB exhibit notably reduced stromal and intratumoral T cell densities: significantly fewer CD3^+^, CD4^+^, and CD8^+^ T cells [66]. FOXP3^+^ T_regs_ were found to be elevated in the high-grade TB cohort. The high-grade TB cohort also exhibited markedly lower densities of B cells, M1 macrophages, and mature dendritic cells, suggesting inhibition of anti-tumor immunity. However, no differences were detected in the density of total macrophages or M2 macrophages, suggesting that both innate and adaptive immune cells, such as M1 macrophages, prevent TB and disease progression in PDAC. They also reported that TCGA’s high-score TB group displayed higher cellular fractions of CAFs and lower proportions of endothelial cells than the low-score TB group. These reports are sufficient to explain the relationship between TB development and antitumor immunity, with CAFs as intermediates. 

## 6. Representative Drivers for TB Development and p-EMT Induction

### 6.1. Hypoxia

**Figure 2:**
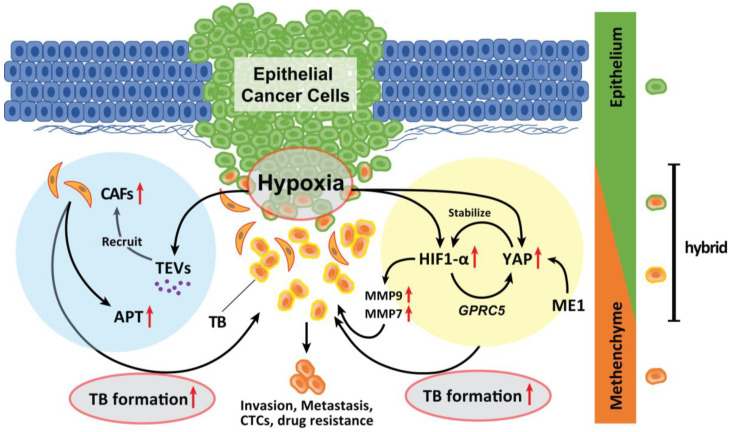
Role of hypoxia in the formation of TB. Hypoxia in advanced TME promotes TB development. Genes whose expression is induced by HIF-1α activation by hypoxia promote EMT. YAP is activated in a hypoxic TME and was abrogated by the knockdown of ME1 which promotes the Warburg effect in cancer cells and induces EMT. YAP activation is a significant factor that promotes the EMT phenotype and is deeply involved in the progression of the tumor. In a hypoxic situation, YAP activation is responsible for the upregulation of *GPRC5A* by binding to HIF-1α. Then, activated YAP also stabilizes HIF-1α and enhances its action. The expression of MMP9 and MMP7 is also upregulated by HIF-1α activation. Moreover, hypoxic TME can induce tumor cells to secrete enhanced amounts of TEVs. The dynamic intercellular crosstalk that is mediated by TEVs mobilizes oncogenic factors, relocalizes CAFs to tumor sites, p-EMT and APT development, and TB formation which sustains cancer progression and metastasis.

Hypoxia in an advanced TME promotes TB development in various types of solid cancer. Hypoxia also promotes the recruitment of CAFs. Genes expressed by hypoxia-inducible factor (HIF)-1α promote EMTs [67]. A recent study has suggested that malic enzyme 1 (ME1) promotes the Warburg effect in cancer cells and induces EMTs in HNSCC cells. Yes-associated proteins (YAP) are activated in the hypoxic TME and abrogated by the knockdown of ME1 [68]. YAP activation promotes EMT and is involved in HNSCC progression [69]. During hypoxia, YAP activation is responsible for the upregulation of *GPRC5A* in combination with HIF-1α [70]. Reciprocally, activated YAP stabilizes HIF-1α and enhances its activity [71]. Inhibition of YAP activation by ME1 knockdown is associated with suppression of ME1 reprogramming of energy metabolism. Moreover, the levels of MMP9 and MMP7 are upregulated by HIF-1α activation; these markers are strong indicators of TB onset in HNSCC and CRC [72,73,74] and are local therapeutic candidates for the prevention of TB development. Therefore, the YAP activation cycle is a key pathway related to TB development induced in response to hypoxia and reprogramming of the energy metabolism (Figure 2). 

Moreover, hypoxic conditions within the TME can induce tumor cells to secrete enhanced amounts of tumor-derived extracellular vesicles (TEVs) [75]. TEVs play critical roles in tumor initiation, progression, and metastasis as vehicles of small molecules [76,77]. The dynamic intercellular crosstalk mediated by TEVs mobilizes oncogenic factors, relocalizes CAFs to tumor sites, and sustains metastasis [78] (Figure 2). Considering that TEVs contribute to the plasticity of cancer cells in multiple stages of cancer progression, TEV-mediated delivery of tumor suppressor cargo has broad possibilities for future clinical applications [79]. 

### 6.2. CAFs

CAFs are key factors in cancer cell invasion because they can remodel the extracellular matrix and provide mechanical propulsive forces that facilitate tumor invasion and metastasis. In vitro, Zhou et al. confirmed that CAFs induced more aggressive carcinoma cell proliferation and human umbilical vascular endothelial cells tube formation, whereas peritumoral fibroblasts strongly promoted the migration of tumor cells, both of which were isolated from patients with HNSCC [80]. Furthermore, it is well known that CAFs secrete molecules involved in tumor invasion, EMTs, and metastasis, including TNF-α, IL-1α/β, IL-33, CCL7, SDF-1, MDNF, type 1 collagen, HGF, IGF2, BMP4, MMPs, PGE2, KGF, activin A, PDGF, and miRNAs [81]. 

In hepatocellular carcinoma, CAF-derived CCL5 (also known as RANTES) promotes metastasis by binding to a specific receptor, CCR5, and inhibiting the ubiquitination and degradation of HIF-1α, maintaining HIF-1α even under normoxia, thereby upregulating the downstream gene *ZEB1* and inducing EMT [82]. In CRC, CCL5 blockade reduced tumor xenograft growth, decreased the migration of tumor cells, reduced liver metastases, and decreased the infiltration of T_regs_ in the tumor [83]. The abnormal expression of CCL5 has been confirmed in many types of tumors, including CRC, breast, lung, ovarian, and prostate cancers [84,85]. Mielcarska et al. reported that CCL5 produced by macrophages can stabilize PD-L1 in CRC cells both in vitro and in vivo. CCL5 induces the formation of the nuclear factor kappa-B (NF-κB) p65/STAT3 complex, which upregulates the promoter of *COP9 signalosome 5* (CSN5). CSN5 stabilizes PD-L1 by regulating its deubiquitination at the cellular level, resulting in increased PD-L1 activity [86]. This may sensitize tumors to immune checkpoint inhibition therapy, even if the cancer cells are resistant to anti-EGFR therapy [87]. Thus, CCL5 blockade is a therapeutic candidate for inhibiting TB development and p-EMT induction in CRC. 

**Figure 3:**
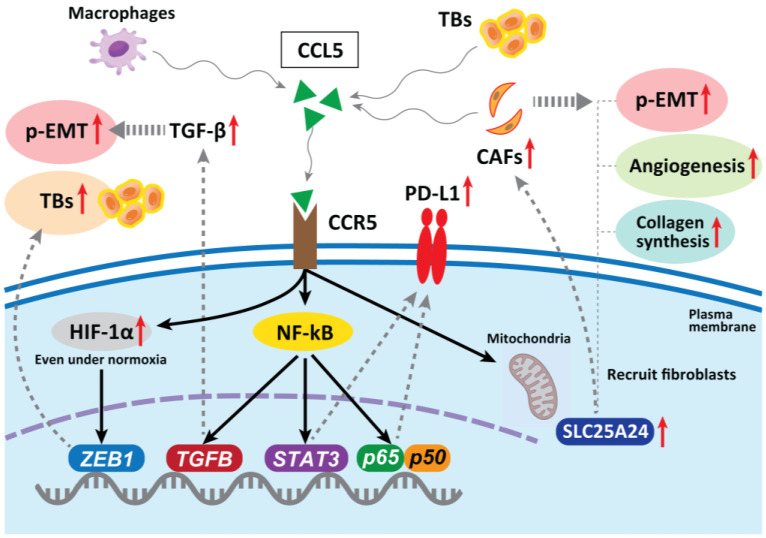
In hepatocellular carcinoma, CAF-derived CCL5 promotes metastasis by binding to specific receptors, CCR5, and stabilizing HIF-1α under normoxia, upregulating one of the EMT genes *ZEB1* and promoting TB development. In CRC, CCL5 blockade reduces tumor growth, decreases migration of tumor cells, reduces metastases, and decreases infiltration of T_regs_ in the tumor. CCL5 can stabilize PD-L1 in vitro and in vivo. CCR5 can also modulate TGF-β activity, which subsequently promotes an EMT. TB cells secrete high levels of CCL5, which recruits fibroblasts through CCR5–*SLC25A24* signaling and leads to the development of a characteristic fibroblast cluster around TB cells at the invasive front of CRC. This further facilitates tumor angiogenesis and collagen synthesis, recruitment of CAFs, and promotes malignant progression. CCR5 can also modulate TGF-β activity, which subsequently promotes an EMT and increases tumor cell migration via the activation of the NF-κB pathway.

CCR5 can modulate TGF-β activity, which subsequently promotes EMT and increases tumor cell migration via activation of the NF-κB pathway [88]. Considering that TB cells exhibit a particular gene expression signature, comprising factors involved in EMT and activated TGF-β signaling [48], the CCR5 contribution to drive p-EMT is promising. Gao et al. revealed that CRC TB cells secrete high levels of CCL5, which recruits CAFs through CCR5–*SLC25A24* signaling and leads to the development of a characteristic fibroblast cluster around TB cells at the invasive front of the tumor. This further facilitates tumor angiogenesis and collagen synthesis and promotes tumor progression [89] (Figure 3). 

In HNSCC, Chuang et al. reported that oral cancer cells with high invasiveness express CCR5 on their surface which regulates the increased migration of tumor cells and metastasis [90]. On the other hand, decreased expression of CCR5 in monocytes from HNSCC patients was reported [91]. Li et al. reported that patients with high CCR5 expression levels had worse overall survival (hazard ratio = 0.59, *p* < 0.001) and worse recurrence-free survival (hazard ratio = 3.27, *p* < 0.001). Moreover, they also observed the upregulation of CCR5 expression is associated with immunomodulators, chemokines, and infiltrating levels of CD4+ T cells, neutrophils, macrophages, and myeloid dendritic cells [92]. Moreover, González-Arriagada et al. reported that significant associations were detected in the relationship between high CCR5 expression and lymph node metastasis (*p* = 0.03), advanced clinical stage (*p* = 0.003), poor differentiation of tumors (*p* = 0.05), and tumor recurrence (*p* = 0.01) [93]. The evidence about the function and contribution of HNSCC CAFs in driving TB development and p-EMT induction is limited and further in-depth investigation will be needed as the above evidence indicates the potential relationship among CCR/CCL5 expression, development of TBs, and p-EMT demonstration. 

### 6.3. Tumor-Associated Macrophages (TAMs)

TAMs have a leading position in the TME and are also key factors in EMT development. In CRC, Trumpi et al. reported that macrophages located around a tumor induce a loss of tight-junction proteins at the tumor cell–cell contacts and cause TBs in the colonosphere bulk [94]. This is because MMP7 is secreted by activation of the NF-κB pathway. Therefore, the macrophage-initiated NF-κB–MMP7 pathway functions as a central player in TB development. 

**Figure 4:**
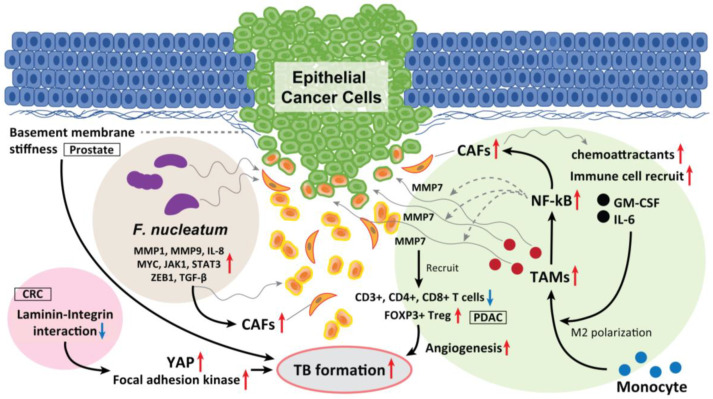
The role of TAM, laminin–integrin interaction, basement membrane stiffness, and *F. nucleatum* as a TB driver. TAMs located in the surrounding of the tumor mass induce loss of tight junction proteins at tumor cell–cell contacts, and cause TB from the colonosphere bulk of CRC. This is due to the MMP7 secretion by activating the NF-κB pathway. Moreover, CAF-derived exosomes serve as chemoattractants, which recruit various immune cells, including monocytes, promoting CRC progression and the release of cancer cell-derived exosomes. In PDAC, high-grade TB cases display lower M1 macrophages in the stroma and increased M2 macrophages in the tumor tissue, and displayed fewer CD3+, CD4+, and CD8+ T cells. Inversely, FOXP3+ T_regs_ were found to be elevated in high-grade TB cases. CAFs also recruit granulocyte-macrophage colony-stimulating factor and IL-6, promote the differentiation of monocytes into M2 macrophages and activate them to release chemokines and exosomes, which promote TB formation. Softening and enhanced remodeling of the basement membrane also promote TB development in stratified epidermis via the activation of focal adhesion kinase and YAP, while stiffening of the basement membrane promotes folding, and the laminin–integrin crosstalk in the basement membrane plays a key role to generate TBs. Moreover, *F. nucleatum* upregulated the expression of p-EMT-related genes in HNSCC cells with an epithelial phenotype. *F. nucleatum*-infected HNSCC cells had upregulated MMP1, MMP9, and IL-8. The expression of cell survival markers MYC, JAK1, and STAT3 and EMT markers ZEB1 and TGF-β were also significantly elevated and promoted TB development. These mediators also recruit CAFs in the TME and promote TB formation.

Moreover, CAF-derived exosomes serve as chemoattractants that recruit various immune cells, including monocytes, thereby promoting CRC progression and the release of cancer-derived exosomes. CAF-derived exosomes containing granulocyte–macrophage colony-stimulating factor and IL-6 promote the differentiation of monocytes into M2 macrophages and activate M2 macrophages to release chemokines and macrophage-derived exosomes, which in turn drive angiogenesis, promoting TB development and metastasis [95] (Figure 4). 

In oral SCC, the existence and roles of M1-like TAMs have been identified and revealed. High infiltration of M1-like TAMs has been identified to be associated with aggressive features of the disease. Xiao et al. demonstrated that exosome-transferred THBS1 polarized macrophages to the M1-like phenotype through p38, Akt, and SAPK/JNK signaling at the early-stage oral SCC [96]. Increased expression of THBS1 could significantly decrease the overall survival of HSNCC patients [97]. Moreover, their RNA sequencing analysis then revealed that M1-like TAMs tightly correlates with the EMT process and cancer-stem characteristics of oral SCC cells: M1-like TAMs could regulate the EMT process of oral SCC cells through the IL6/Jak/Stat3 signaling pathway, which could subsequently promote the transcription and expression of THBS1 [97]. 

### 6.4. Laminin-5γ2 (LN-5γ2) and Integrin β1

The glycoprotein LN-5γ2 has recently become a focus of increased interest and investigation as a marker of invasion in gastrointestinal malignancies. In oral SCC, Peixoto da-Silva et al. suggested that heterogeneous LN-5γ2 chain expression in the invasive front of the tumor mediates the acquisition of the migrating and invading epithelial cell phenotype [98]. Among the interactions between a tumor and the surrounding stroma in oral SCC, Marangon Junior et al. reported that high-grade TB was associated with a higher expression of LN-5γ2, which is a cell–extracellular matrix adhesion molecule [99]. Zhou et al. reported that the interaction between LN-5γ2 and integrin β1 in CRC promoted TB development via focal adhesion kinase and YAP activation. This induces the nuclear translocation of YAP/TAZ, which consequently promotes tumor growth, EMTs, and TB development by regulating the transcription of downstream genes. Thus, high expression levels of LN-5γ2 and integrin β1 may indirectly improve the diagnostic sensitivity for occult TB development. They also found that a natural medicinal monomer, cucurbitacin B, inhibits the interaction between LN-5γ2 and integrin β1, inactivates YAP, and blocks TB development [100]. 

Fiore et al. reported that softening and enhanced remodeling of the basement membrane also promotes TB in the stratified epidermis while the stiffening of the basement membrane promotes folding [101]. Moreover, Wang et al. observed prominent TB formation in β-integrin–depleted prostate cancer spheroids in regions of a discontinuous laminin-332 layer, which was not observed in the controls. They concluded that the loss of integrin β4 expression generates laminin-332 continuity gaps, through which basal cells exit the spheroid, promoting the progression of high-grade prostatic intraepithelial neoplasia [102]. Thus, laminin–integrin interaction and basement membrane stiffness can also play a key role in promoting TBs, EMT/p-EMT, invasion, and metastasis (Figure 4). These physical properties of epithelial cancer tissue also affect the fate of tumor control due to TB development.

### 6.5. Fusobacterium nucleatum (F. nucleatum)

*F. nucleatum* has pathogenic effects on HNSCC and CRC [103]. *F. nucleatum* is detected more frequently in deeper areas of cancer tissues than in healthy subjects [104,105]. *F. nucleatum* is also frequently detected in CRC tissues and is directly involved in CRC development [106]. In addition, Harrandah et al. reported enhanced expression of three oncogenes (*STAT3*, *JAK1*, and *MYC*) and EMT markers in *F. nucleatum*-infected oral cancer cell lines. *F. nucleatum* can enhance MMP1, MMP9, and IL-8 expression and cancer cell invasiveness. They also upregulate the expression of p-EMT-related genes in oral SCC cells with an epithelial phenotype but not in those with a p-EMT or EMT phenotype. 

In HNSCC, as we previously summarized periodontitis-related bacterial contributions to gingival carcinogenesis and gingival SCC progression [107], *F. nucleatum* is one of the bacteria deeply involved in the development of oral cancer [107]. Harrandah et al. reported that *F. nucleatum*–polyinfected (c.f. *Porphyromonas gingivalis*) oral SCC cells showed synergistically upregulated expression of MMP1, MMP9, and IL-8; the expression of cell survival markers MYC, JAK1, and STAT3, and the EMT markers ZEB1 and TGF-β were significantly elevated (Figure 4) [107,108]. Moreover, there is definite evidence of intratumoral microbiota that is highly organized in micro niches with immune and epithelial cell functions that support cancer progression. Epithelial cancer cells that are infected with *F. nucleatum* invade their surrounding TME as single cells and recruit myeloid cells to bacterial regions promoting transcriptional changes in epithelial cancer cells that facilitate invasion into the deeper [109]. Taken together, *F. nucleatum* drives TB development and EMT/p-EMT induction in the TME of oral cancer, which is promoted by polyinfection.

### 6.6. Human Papilloma Virus (HPV) Status

In HNSCC, HPV infection must be considered; however, there is no clear consensus that has yet been reached on the relationship between TB development and HPV infection. In vulvovaginal SCC, HPV-independent carcinomas are more likely to be moderately and poorly differentiated, with an intermediate-to-high TB status [110]. Since the expression of p16 correlates with high TWIST, SNAIL, and SLUG expression in oropharyngeal SCC [111], HPV infection itself may act as a TB driver. However, Prell et al. reported that the upregulation of p16^INK4a^ may not be a strict requirement for TB development in CRC, suggesting that there is no correlation between the degree of p16 expression and TB development [112]. Further analyses of how HPV infection is related to TB development and p-EMT induction in HPV-associated HNSCCs (including the dynamics modulations of E5, E6, and E7 kinases) are required in the future. 

### 6.7. Methylthioadenosine Phosphorylase (MTAP)

MTAP is a rate-limiting enzyme in the methionine salvage pathway, which recycles one carbon unit lost during polyamine synthesis into the methionine cycle. The *MTAP* gene, which is located at the chromosomal locus 9p21, is deleted in many human cancers because of its proximity to the tumor suppressor gene *cyclin-dependent kinase inhibitor 2A*. When the level of MTAP increases, it prevents the combination of cyclin D1 and CDK4, cell cycle arrest in the G1 phase, and the inhibition of cell proliferation. MTAP deficiency commonly occurs in hematological malignancies and various solid tumors, suggesting that MTAP may play a tumor-suppressing role in these types of cancer [113]. However, in CRC, MTAP expression is upregulated by LEF/TCF/β-catenin in parallel with tumor progression and cell dedifferentiation [114,115]. Amano et al. reported that concomitant nuclear and cytoplasmic expression of MTAP in OSCC is associated with a high TB score and might promote tumor aggressiveness through its activity in the methionine salvage pathway. In oral SCC, cytoplasmic MTAP expression is observed from an early stage of oncogenesis and persists throughout the progression of the disease. Both nuclear and cytoplasmic MTAP expressions are associated with an aggressive invasion pattern and EMT [116]. Thus, high MTAP expression levels in both may indicate the p-EMT/EMT process in oral SCC, and finding the relationship between and mechanism among MTAP, the LEF/TCF/β-catenin pathway, TB development, and their intermediates in the TME are required. 

Representative evidence for TB drivers and p-EMT induction has been mentioned above; other well-described candidates for the TB driver are summarized in Table 1 [30,117,118,119,120]. 

## 7. Conclusions and Future Perspective

Understanding TME and intratumoral heterogeneity is a major challenge in oncology. The TME has an important influence on the biological behavior of cancer, and tumor–host interactions are essential for the invasion and metastasis of various cancers. In this review, we summarized the crosstalk in the TME, especially regarding TB formation and tumor EMT status, focusing on p-EMT. To date, several studies on TB drivers have been conducted, and important evidence regarding these relationships has been revealed for every type of solid cancer. However, as in the previous section and Table 1, in-depth research works about TB drivers are largely from the field of CRC. This discrepancy might be due to the rarity of the HNSCC cases compared with CRC. Histologically, they are both of epithelial origin and several genes in HNSCC and CRC are commonly altered (e.g., *TP53, EGFR, PIK3CA, PTEN*, etc.) [121,122]. In addition, it is well-known that *F. nucleatum* exists in both the oral cavity and colorectal tract, and in both fields, it plays key roles in carcinogenesis and progression [109,123]. In parallel, there is evidence that the oral and gastrointestinal mucosae are microbiologically and immunologically connected [124]. Thus, from the similarity of the states in both lesions, there should be common mechanisms for the development of TBs and induction of p-EMT between HNSCC and CRC. 

Therefore, the next step is to reveal whether the evidence and relationship can be adapted to HNSCC and to establish its organized relationship. Specifically, as an example, using tissue samples, a combination of successive single cell-RNA sequences can comprehensively confirm the differences in the gene amplifications and the patterns in the TME between HNSCC and CRC. Understanding the mechanism of TB onset and induction of p-EMTs may lead to the development of novel diagnostic and prognostic methods and targeted therapies for the prevention of invasion and metastasis in epithelial cancer (e.g., targeting CAFs and their exosomes/extracellular vehicles/microRNAs, angiogenesis, and reoxygenation in the TME, development of antitumor agent delivery systems, and viral control, etc.). These endeavors in TB studies are underway. 

## Figures and Tables

**Table 1:** Other evidence of TB drivers.

TB Driver	Mechanism	Cancer Type	Year	Reference
COL4A1/COL13A1	Activation of intracellular AKT pathway leads to an E/N-cadherin switch	Urothelial carcinoma of bladder	2017	[31]
SerpinE1 (known as plasminogen activator inhibitor type 1)	Regulation of the plasminogen activator system	HNSCC	2019	[32]
c-MET	Upregulation of MET transcription	CRC	2016	[30]
Tenascin-C	Tenascin-C induces cancer cell EMT-like change	CRC	2018	[117]
SREBP1	Upregulation of MMP7expression and NF-κB pathway activation	CRC	2019	[118]
Increased tumor stroma Percentage and LDH-5	Decrease CD3+ lymphocyte stromal density	CRC	2019	[119]
Thymosin β4/β10	Modulation of cytoskeleton organization	CRC	2021	[120]

## References

[1] Ueno H., Murphy J., Jass J.R., Mochizuki H., Talbot I.C. (2002). Tumourbudding’as an index to estimate the potential of aggressiveness in rectal cancer. Histopathology.

[2] Almangush A., Pirinen M., Heikkinen I., Mäkitie A.A., Salo T., Leivo I. (2018). Tumour budding in oral squamous cell carcinoma: A meta-analysis. Br. J. Cancer.

[3] Zhu Y., Liu H., Xie N., Liu X., Huang H., Wang C., Hou J. (2019). Impact of tumor budding in head and neck squamous cell carcinoma: A meta-analysis. Head Neck.

[4] Okuyama K., Fukushima H., Naruse T., Yanamoto S., Tsuchihashi H., Umeda M. (2019). CD44 Variant 6 Expression and Tumor Budding in the Medullary Invasion Front of Mandibular Gingival Squamous Cell Carcinoma Are Predictive Factors for Cervical Lymph Node Metastasis. Pathol. Oncol. Res..

[5] Attramadal C.G., Kumar S., Boysen M.E., Dhakal H.P., Nesland J.M., Bryne M. (2015). Tumor Budding, EMT and Cancer Stem Cells in T1-2/N0 Oral Squamous Cell Carcinomas. Anticancer Res..

[6] Dolens E.D.S., Dourado M.R., Almangush A., Salo T.A., Gurgel Rocha C.A., da Silva S.D., Brennan P.A., Coletta R.D. (2021). The Impact of Histopathological Features on the Prognosis of Oral Squamous Cell Carcinoma: A Comprehensive Review and Meta-Analysis. Front. Oncol..

[7] Karjol U., Jonnada P., Annavarjula V., Cherukuru S., Chandranath A., Anwar A. (2020). Prognostic Role of Tumor Budding in Carcinoma Tongue: A Systemic Review and Meta-Analysis. Cureus.

[8] Lugli A., Karamitopoulou E., Zlobec I. (2012). Tumour budding: A promising parameter in colorectal cancer. Br. J. Cancer.

[9] Rogers A.C., Winter D.C., Heeney A., Gibbons D., Lugli A., Puppa G., Sheahan K. (2016). Systematic review and meta-analysis of the impact of tumour budding in colorectal cancer. Br. J. Cancer.

[10] Beaton C., Twine C.P., Williams G.L., Radcliffe A.G. (2013). Systematic review and meta-analysis of histopathological factors influencing the risk of lymph node metastasis in early colorectal cancer. Color. Dis..

[11] Niwa Y., Yamada S., Koike M., Kanda M., Fujii T., Nakayama G., Sugimoto H., Nomoto S., Fujiwara M., Kodera Y. (2014). Epithelial to Mesenchymal Transition Correlates with Tumor Budding and Predicts Prognosis in Esophageal Squamous Cell Carcinoma. J. Surg. Oncol..

[12] Brown M., Sillah K., Griffiths E.A., Swindell R., West C.M., Page R.D., Welch I.M., Pritchard S.A. (2010). Tumour budding and a low host inflammatory response are associated with a poor prognosis in oesophageal and gastro-oesophageal junction cancers. Histopathology.

[13] Koelzer V.H., Langer R., Zlobec I., Lugli A. (2014). Tumor budding in upper gastrointestinal carcinomas. Front. Oncol..

[14] Xiang Z., He Q., Huang L., Xiong B., Xiang Q. (2022). Breast Cancer Classification Based on Tumor Budding and Stem Cell-Related Signatures Facilitate Prognosis Evaluation. Front. Oncol..

[15] Masuda R., Kijima H., Imamura N., Aruga N., Nakamura Y., Masuda D., Takeichi H., Kato N., Nakagawa T., Tanaka M. (2012). Tumor budding is a significant indicator of a poor prognosis in lung squamous cell carcinoma patients. Mol. Med. Rep..

[16] Kadota K., Yeh Y.C., Villena-Vargas J., Cherkassky L., Drill E.N., Sima C.S., Jones D.R., Travis W.D., Adusumilli P.S. (2015). Tumor Budding Correlates with the Protumor Immune Microenvironment and Is an Independent Prognostic Factor for Recurrence of Stage I Lung Adenocarcinoma. Chest.

[17] Budau K.L., Sigel C.S., Bergmann L., Lüchtenborg A.M., Wellner U., Schilling O., Werner M., Tang L., Bronsert P. (2022). Prognostic Impact of Tumor Budding in Intrahepatic Cholangiocellular Carcinoma. J. Cancer.

[18] Argon A., Öz Ö., Kebat T.A. (2023). Evaluation and prognostic significance of tumor budding in pancreatic ductal adenocarcinomas. Indian J. Pathol. Microbiol..

[19] Szalai L., Jakab Á., Kocsmár I., Szirtes I., Kenessey I., Szijártó A., Schaff Z., Kiss A., Lotz G., Kocsmár É. (2022). Prognostic Ability of Tumor Budding Outperforms Poorly Differentiated Clusters in Gastric Cancer. Cancers.

[20] Le T.M., Nguyen H.D.T., Lee E., Lee D., Choi Y.S., Cho J., Park N.J., Han H.S., Chong G.O. (2022). Transcriptomic Immune Profiles Can Represent the Tumor Immune Microenvironment Related to the Tumor Budding Histology in Uterine Cervical Cancer. Genes.

[21] Choi Y., Park N.J., Le T.M., Lee E., Lee D., Nguyen H.D.T., Cho J., Park J.Y., Han H.S., Chong G.O. (2022). Immune Pathway and Gene Database (IMPAGT) Revealed the Immune Dysregulation Dynamics and Overactivation of the PI3K/Akt Pathway in Tumor Buddings of Cervical Cancer. Curr. Issues Mol. Biol..

[22] Ocal I., Guzelis I. (2022). Tumor budding is a valuable prognostic parameter in endometrial carcinomas. Indian J. Pathol. Microbiol..

[23] Yang Y., Xu H., Zhu H., Yuan D., Zhang H., Liu Z., Zhao F., Liang G. (2022). EPDR1 levels and tumor budding predict and affect the prognosis of bladder carcinoma. Front. Oncol..

[24] Lino-Silva L.S., Zepeda-Najar C., Caro-Sánchez C.H., Herrera-Gómez Á., Salcedo-Hernández R.A. (2022). Prognostic significance of tumor budding in melanoma. Melanoma Res..

[25] Ling Z., Cheng B., Tao X. (2020). Epithelial-to-mesenchymal transition in oral squamous cell carcinoma: Challenges and opportunities. Int. J. Cancer.

[26] Grigore A.D., Jolly M.K., Jia D., Farach-Carson M.C., Levine H. (2016). Tumor budding: The name is EMT. Partial EMT. J. Clin. Med..

[27] Wang C., Huang H., Huang Z., Wang A., Chen X., Huang L., Zhou X., Liu X. (2011). Tumor budding correlates with poor prognosis and epithelial- mesenchymal transition in tongue squamous cell carcinoma. J. Oral Pathol. Med..

[28] Zlobec I., Lugli A. (2010). Epithelial mesenchymal transition and tumor budding in aggressive colorectal cancer: Tumor budding as oncotarget. Oncotarget.

[29] Galván J.A., Zlobec I., Wartenberg M., Lugli A., Gloor B., Perren A., Karamitopoulou E. (2015). Expression of E-cadherin repressors SNAIL, ZEB1 and ZEB2 by tumour and stromal cells influences tumour-budding phenotype and suggests heterogeneity of stromal cells in pancreatic cancer. Br. J. Cancer.

[30] Bradley C.A., Dunne P.D., Bingham V., McQuaid S., Khawaja H., Craig S., James J., Moore W.L., McArt D.G., Lawler M. (2016). Transcriptional upregulation of c-MET is associated with invasion and tumor budding in colorectal cancer. Oncotarget.

[31] Miyake M., Hori S., Morizawa Y., Tatsumi Y., Toritsuka M., Ohnishi S., Shimada K., Furuya H., Khadka V.S., Deng Y. (2017). Collagen type IV alpha 1 (COL4A1) and collagen type XIII alpha 1 (COL13A1) produced in cancer cells promote tumor budding at the invasion front in human urothelial carcinoma of the bladder. Oncotarget.

[32] Arroyo-Solera I., Pavón M.Á., Leon X., Lopez M., Gallardo A., Céspedes M.V., Casanova I., Pallares V., López-Pousa A., Mangues M.A. (2019). Effect of serpinE1 overexpression on the primary tumor and lymph node, and lung metastases in head and neck squamous cell carcinoma. Head Neck.

[33] Xie N., Wang C., Zhuang Z., Hou J., Liu X., Wu Y., Liu H., Huang H. (2016). Decreased miR-320a promotes invasion and metastasis of tumor budding cells in tongue squamous cell carcinoma. Oncotarget.

[34] Parikh A.S., Puram S.V., Faquin W.C., Richmon J.D., Emerick K.S., Deschler D.G., Varvares M.A., Tirosh I., Bernstein B.E., Lin D.T. (2019). Immunohistochemical quantification of partial-EMT in oral cavity squamous cell carcinoma primary tumors is associated with nodal metastasis. Oral Oncol..

[35] Xie N., Wang C., Liu X., Li R., Hou J., Chen X., Huang H. (2015). Tumor budding correlates with occult cervical lymph node metastasis and poor prognosis in clinical early-stage tongue squamous cell carcinoma. J. Oral Pathol. Med..

[36] Pastushenko I., Brisebarre A., Sifrim A., Fioramonti M., Revenco T., Boumahdi S., Van Keymeulen A., Brown D., Moers V., Lemaire S. (2018). Identification of the tumour transition states occurring during EMT. Nature.

[37] Pastushenko I., Blanpain C. (2019). EMT transition states during tumor progression and metastasis. Trends Cell Biol..

[38] Sarioglu S., Acara C., Akman F.C., Dag N., Ecevit C., Ikiz A.O., Cetinayak O.H., Ada E. (2010). Tumor budding as a prognostic marker in laryngeal carcinoma. Pathol. Res. Pract..

[39] Gonzalez-Guerrero M., Martínez-Camblor P., Vivanco B., Fernández-Vega I., Munguía-Calzada P., Gonzalez-Gutierrez M.P., Rodrigo J.P., Galache C., Santos-Juanes J. (2017). The adverse prognostic effect of tumor budding on the evolution of cutaneous head and neck squamous cell carcinoma. J. Am. Acad. Dermatol..

[40] De Smedt L., Palmans S., Andel D., Govaere O., Boeckx B., Smeets D., Galle E., Wouters J., Barras D., Suffiotti M. (2017). Expression profiling of budding cells in colorectal cancer reveals an EMT-like phenotype and molecular subtype switching. Br. J. Cancer.

[41] Mannelli G., Gallo O. (2012). Cancer stem cells hypothesis and stem cells in head and neck cancers. Cancer Treat. Rev..

[42] Duester G. (2008). Retinoic acid synthesis and signaling during early organogenesis. Cell.

[43] Luo W.R., Gao F., Li S.Y., Yao K.T. (2012). Tumour budding and the expression of cancer stem cell marker aldehyde dehydrogenase 1 in nasopharyngeal carcinoma. Histopathology.

[44] Marangon Junior H., Melo V.V.M., Caixeta Â.B., Souto G.R., Souza P.E.A., de Aguiar M.C.F., Horta M.C.R. (2019). Immunolocalization of cancer stem cells marker ALDH1 and its association with tumor budding in oral squamous cell carcinoma. Head Neck Pathol.

[45] Boxberg M., Götz C., Haidari S., Dorfner C., Jesinghaus M., Drecoll E., Boskov M., Wolff K.D., Weichert W., Haller B. (2018). Immunohistochemical expression of CD44 in oral squamous cell carcinoma in relation to histomorphological parameters and clinicopathological factors. Histopathology.

[46] Mäkitie A.A., Almangush A., Rodrigo J.P., Ferlito A., Leivo I. (2019). Hallmarks of cancer: Tumor budding as a sign of invasion and metastasis in head and neck cancer. Head Neck.

[47] Jensen D.H., Dabelsteen E., Specht L., Fiehn A.M.K., Therkildsen M.H., Jønson L., Vikesaa J., Nielsen F.C., Von Buchwald C. (2015). Molecular profiling of tumour budding implicates TGF beta-mediated epithelial-mesenchymal transition as a therapeutic target in oral squamous cell carcinoma. J. Pathol..

[48] Bronsert P., Enderle-Ammour K., Bader M., Timme S., Kuehs M., Csanadi A., Kayser G., Kohler I., Bausch D., Hoeppner J. (2014). Cancer cell invasion and EMT marker expression: A three-dimensional study of the human cancer-host interface. J. Pathol..

[49] Sharaf K., Lechner A., Haider S.P., Wiebringhaus R., Walz C., Kranz G., Canis M., Haubner F., Gires O., Baumeister P. (2021). Discrimination of Cancer Stem Cell Markers ALDH1A1, BCL11B, BMI-1, and CD44 in Different Tissues of HNSCC Patients. Curr. Oncol..

[50] Jakob M., Sharaf K., Schirmer M., Leu M., Küffer S., Bertlich M., Ihler F., Haubner F., Canis M., Kitz J. (2021). Role of cancer stem cell markers ALDH1, BCL11B, BMI-1, and CD44 in the prognosis of advanced HNSCC. Strahlenther. Onkol..

[51] Wang W., Xie N., Yi C., Zhang M., Xiong G., Xu X., Hou J., Wang C. (2022). Prognostic and clinicopathological significance of cytocapsular tubes in oral squamous cell carcinoma. J. Oral Pathol. Med..

[52] Puram S.V., Parikh A.S., Tirosh I. (2018). Single cell RNA- seq highlights a role for a partial EMT in head and neck cancer. Mol. Cell. Oncol..

[53] Puram S.V., Tirosh I., Parikh A.S., Patel A.P., Yizhak K., Gillespie S., Rodman C., Luo C.L., Mroz E.A., Emerick K.S. (2017). Single-Cell Transcriptomic Analysis of Primary and Metastatic Tumor Ecosystems in Head and Neck Cancer. Cell.

[54] Bolós V., Peinado H., Pérez-Moreno M.A., Fraga M.F., Esteller M., Cano A. (2003). The transcription factor Slug represses E-cadherin expression and induces epithelial to mesenchymal transitions: A comparison with Snail and E47 repressors. J. Cell Sci..

[55] Yang J., Antin P., Berx G., Blanpain C., Brabletz T., Bronner M., Campbell K., Cano A., Casanova J., Christofori G. (2020). Guidelines and definitions for research on epithelial-mesenchymal transition. Nat. Rev. Mol. Cell Biol..

[56] Okuyama K., Suzuki K., Yanamoto S., Naruse T., Tsuchihashi H., Yamashita S., Umeda M. (2018). Anaplastic transition within the cancer microenvironment in early-stage oral tongue squamous cell carcinoma is associated with local recurrence. Int. J. Oncol..

[57] Kohler I., Bronsert P., Timme S., Werner M., Brabletz T., Hopt U.T., Schilling O., Bausch D., Keck T., Wellner U.F. (2015). Detailed analysis of epithelial-mesenchymal transition and tumor budding identifies predictors of long-term survival in pancreatic ductal adenocarcinoma. J. Gastroenterol. Hepatol..

[58] Saini S., Tulla K., Maker A.V., Burman K.D., Prabhakar B.S. (2018). Therapeutic advances in anaplastic thyroid cancer: A current perspective. Mol. Cancer.

[59] O’Neill J.P., Shaha A.R. (2013). Anaplastic thyroid cancer. Oral Oncol..

[60] Perrier N.D., Brierley J.D., Tuttle R.M. (2018). Differentiated and anaplastic thyroid carcinoma: Major changes in the American Joint Committee on Cancer eighth edition cancer staging manual. CA Cancer J. Clin..

[61] Meyer S.N., Galván J.A., Zahnd S., Sokol L., Dawson H., Lugli A., Zlobec I. (2019). Co-expression of cytokeratin and vimentin in colorectal cancer highlights a subset of tumor buds and an atypical cancer-associated stroma. Hum. Pathol..

[62] Wartenberg M., Zlobec I., Perren A., Koelzer V.H., Gloor B., Lugli A., Eva K. (2015). Accumulation of FOXP3+T-cells in the tumor microenvironment is associated with an epithelial-mesenchymal-transition-type tumor budding phenotype and is an independent prognostic factor in surgically resected pancreatic ductal adenocarcinoma. Oncotarget.

[63] Wartenberg M., Cibin S., Zlobec I., Vassella E., Eppenberger-Castori S., Terracciano L., Eichmann M.D., Worni M., Gloor B., Perren A. (2018). Integrated Genomic and Immunophenotypic Classification of Pancreatic Cancer Reveals Three Distinct Subtypes with Prognostic/Predictive Significance. Clin. Cancer Res..

[64] Chen S.J., Liu H., Liao C.T., Huang P.J., Huang Y., Hsu A., Tang P., Chang Y.S., Chen H.C., Yen T.C. (2015). Ultra-deep targeted sequencing of advanced oral squamous cell carcinoma identifies a mutation-based prognostic gene signature. Oncotarget.

[65] Moreira A., Poulet A., Masliah-Planchon J., Lecerf C., Vacher S., Chérif L.L., Dupain C., Marret G., Girard E., Syx L. (2021). Prognostic value of tumor mutational burden in patients with oral cavity squamous cell carcinoma treated with upfront surgery. ESMO Open.

[66] Sadozai H., Acharjee A., Gruber T., Gloor B., Karamitopoulou E. (2021). Pancreatic Cancers with High Grade Tumor Budding Exhibit Hallmarks of Diminished Anti-Tumor Immunity. Cancers.

[67] Wong C.C., Kai A.K., Ng I.O. (2014). The impact of hypoxia in hepatocellular carcinoma metastasis. Front. Med..

[68] Nakashima C., Kirita T., Yamamoto K., Mori S., Luo Y., Sasaki T., Fujii K., Ohmori H., Kawahara I., Mori T. (2020). Malic Enzyme 1 Is Associated with Tumor Budding in Oral Squamous Cell Carcinomas. Int. J. Mol. Sci..

[69] Nakashima C., Yamamoto K., Kishi S., Sasaki T., Ohmori H., Fujiwara-Tani R., Mori S., Kawahara I., Nishiguchi Y., Mori T. (2020). Clostridium perfringens enterotoxin induces claudin-4 to activate YAP in oral squamous cell carcinomas. Oncotarget.

[70] Greenhough A., Bagley C., Heesom K.J., Gurevich D.B., Gay D., Bond M., Collard T.J., Paraskeva C., Martin P., Sansom O.J. (2018). Cancer cell adaptation to hypoxia involves a HIF-GPRC5A-YAP axis. EMBO Mol. Med..

[71] Zhang X., Li Y., Ma Y., Yang L., Wang T., Meng X., Zong Z., Sun X., Hua X., Li H. (2018). Yes-associated protein (YAP) binds to HIF-1α and sustains HIF-1α protein stability to promote hepatocellular carcinoma cell glycolysis under hypoxic stress. J. Exp. Clin. Cancer Res..

[72] Guzińska-Ustymowicz K. (2006). MMP-9 and cathepsin B expression in tumor budding as an indicator of a more aggressive phenotype of colorectal cancer (CRC). Anticancer Res..

[73] Masaki T., Matsuoka H., Sugiyama M., Abe N., Goto A., Sakamoto A., Atomi Y. (2001). Matrilysin (MMP-7) as a significant determinant of malignant potential of early invasive colorectal carcinomas. Br. J. Cancer.

[74] Nascimento G.J.F.D., Silva L.P.D., Matos F.R., Silva T.A.D., Medeiros S.R.B., Souza L.B., Freitas R.A. (2020). Polymorphisms of matrix metalloproteinase-7 and -9 are associated with oral tongue squamous cell carcinoma. Braz. Oral Res..

[75] King H.W., Michael M.Z., Gleadle J.M. (2012). Hypoxic enhancement of exosome release by breast cancer cells. BMC Cancer.

[76] Montecalvo A., Larregina A.T., Shufesky W.J., Beer Stolz D., Sullivan M.L., Karlsson J.M., Baty C.J., Gibson G.A., Erdos G., Wang Z. (2012). Mechanism of transfer of functional microRNAs between mouse dendritic cells via exosomes. Blood.

[77] Li J., Zhang Y., Liu Y., Dai X., Li W., Cai X., Yin Y., Wang Q., Xue Y., Wang C. (2013). Microvesicle-mediated transfer of microRNA-150 from monocytes to endothelial cells promotes angiogenesis. J. Biol. Chem..

[78] Quante M., Tu S.P., Tomita H., Gonda T., Wang S.S., Takashi S., Baik G.H., Shibata W., DiPrete B., Betz K.S. (2011). Bone marrow-derived myofibroblasts contribute to the mesenchymal stem cell niche and promote tumor growth. Cancer Cell.

[79] Wang H.X., Gires O. (2019). Tumor-derived extracellular vesicles in breast cancer: From bench to bedside. Cancer Lett..

[80] Zhou J., Schwenk-Zieger S., Kranz G., Walz C., Klauschen F., Dhawan S., Canis M., Gires O., Haubner F., Baumeister P. (2022). Isolation and characterization of head and neck cancer-derived peritumoral and cancer-associated fibroblasts. Front. Oncol..

[81] Wang J., Min A., Gao S., Tang Z. (2014). Genetic regulation and potentially therapeutic application of cancer-associated fibroblasts in oral cancer. J. Oral Pathol. Med..

[82] Xu H., Zhao J., Li J., Zhu Z., Cui Z., Liu R., Lu R., Yao Z., Xu Q. (2022). Cancer associated fibroblast-derived CCL5 promotes hepatocellular carcinoma metastasis through activating HIF1α/ZEB1 axis. Cell Death Dis..

[83] Chang L.Y., Lin Y.C., Mahalingam J., Huang C.T., Chen T.W., Kang C.W., Peng H.M., Chu Y.Y., Chiang J.M., Dutta A. (2012). Tumor-derived chemokine CCL5 enhances TGF-β-mediated killing of CD8(+) T cells in colon cancer by T-regulatory cells. Cancer Res..

[84] Aldinucci D., Borghese C., Casagrande N. (2020). The CCL5/CCR5 Axis in Cancer Progression. Cancers.

[85] Aldinucci D., Colombatti A. (2014). The inflammatory chemokine CCL5 and cancer progression. Mediat. Inflamm..

[86] Mielcarska S., Kula A., Dawidowicz M., Kiczmer P., Chrabańska M., Rynkiewicz M., Wziątek-Kuczmik D., Świętochowska E., Waniczek D. (2022). Assessment of the RANTES Level Correlation and Selected Inflammatory and Pro-Angiogenic Molecules Evaluation of Their Influence on CRC Clinical Features: A Preliminary Observational Study. Medicina.

[87] Okuyama K., Yanamoto S. (2022). TMEM16A as a potential treatment target for head and neck cancer. J. Exp. Clin. Cancer Res..

[88] Liu J., Wang C., Ma X., Tian Y., Wang C., Fu Y., Luo Y. (2019). High expression of CCR5 in melanoma enhances epithelial-mesenchymal transition and metastasis via TGFβ1. J. Pathol..

[89] Gao L.F., Zhong Y., Long T., Wang X., Zhu J.X., Wang X.Y., Hu Z.Y., Li Z.G. (2022). Tumor bud-derived CCL5 recruits fibroblasts and promotes colorectal cancer progression via CCR5-SLC25A24 signaling. J. Exp. Clin. Cancer Res..

[90] Chuang J.Y., Yang W.H., Chen H.T. (2009). CCL5/CCR5 axis promotes the motility of human oral cancer cells. J. Cell Physiol..

[91] Lang S., Lauffer L., Clausen C., Löhr I., Schmitt B., Hölzel D., Wollenberg B., Gires O., Kastenbauer E., Zeidler R. (2003). Impaired monocyte function in cancer patients: Restoration with a cyclooxygenase-2 inhibitor. FASEB J..

[92] Li C., Chen S., Liu C., Mo C., Gong W., Hu J., He M., Xie L., Hou X., Tang J. (2022). CCR5 as a prognostic biomarker correlated with immune infiltrates in head and neck squamous cell carcinoma by bioinformatic study. Hereditas.

[93] González-Arriagada W.A., Lozano-Burgos C., Zúñiga-Moreta R., González-Díaz P., Coletta R.D. (2018). Clinicopathological significance of chemokine receptor (CCR1, CCR3, CCR4, CCR5, CCR7 and CXCR4) expression in head and neck squamous cell carcinomas. J. Oral Pathol. Med..

[94] Trumpi K., Frenkel N., Peters T., Korthagen N.M., Jongen J.M., Raats D., van Grevenstein H., Backes Y., Moons L.M., Lacle M.M. (2018). Macrophages induce “budding” in aggressive human colon cancer subtypes by protease-mediated disruption of tight junctions. Oncotarget.

[95] Wang M., Su Z., Amoah Barnie P. (2020). Crosstalk among colon cancer-derived exosomes, fibroblast-derived exosomes, and macrophage phenotypes in colon cancer metastasis. Int. Immunopharmacol..

[96] Xiao M., Zhang J., Chen W., Chen W. (2018). M1-like tumor-associated macrophages activated by exosome-transferred THBS1 promote malignant migration in oral squamous cell carcinoma. J. Exp. Clin. Cancer Res..

[97] You Y., Tian Z., Du Z., Wu K., Xu G., Dai M., Wang Y., Xiao M. (2022). M1-like tumor-associated macrophages cascade a mesenchymal/stem-like phenotype of oral squamous cell carcinoma via the IL6/Stat3/THBS1 feedback loop. J. Exp. Clin. Cancer Res..

[98] Peixoto da-Silva J., Lourenço S., Nico M., Silva F.H., Martins M.T., Costa-Neves A. (2012). Expression of laminin-5 and integrins in actinic cheilitis and superficially invasive squamous cell carcinomas of the lip. Pathol. Res. Pract..

[99] Marangon Junior H., Rocha V.N., Leite C.F., de Aguiar M.C.F., Souza P.E.A., Horta M.C.R. (2014). Laminin-5 gamma 2 chain expression is associated with intensity of tumor budding and density of stromal myofibroblasts in oral squamous cell carcinoma. J. Oral Pathol. Med..

[100] Zhou B., Zong S., Zhong W., Tian Y., Wang L., Zhang Q., Zhang R., Li L., Wang W., Zhao J. (2020). Interaction between laminin-5γ2 and integrin β1 promotes the tumor budding of colorectal cancer via the activation of Yes-associated proteins. Oncogene.

[101] Fiore V.F., Krajnc M., Quiroz F.G., Levorse J., Pasolli H.A., Shvartsman S.Y., Fuchs E. (2020). Mechanics of a multilayer epithelium instruct tumour architecture and function. Nature.

[102] Wang M., Nagle R.B., Knudsen B.S., Rogers G.C., Cress A.E. (2017). A basal cell defect promotes budding of prostatic intraepithelial neoplasia. J. Cell Sci..

[103] Gholizadeh P., Eslami H., Kafil H.S. (2017). Carcinogenesis mechanisms of *Fusobacterium nucleatum*. Biomed. Pharmacother..

[104] Al-Hebshi N.N., Nasher A.T., Maryoud M.Y., Homeida H.E., Chen T., Idris A.M., Johnson N.W. (2017). Inflammatory bacteriome featuring *Fusobacterium nucleatum* and *Pseudomonas aeruginosa* identified in association with oral squamous cell carcinoma. Sci. Rep..

[105] Shao W., Fujiwara N., Mouri Y., Kisoda S., Yoshida K., Yoshida K., Yumoto H., Ozaki K., Ishimaru N., Kudo Y. (2021). Conversion from epithelial to partial-EMT phenotype by *Fusobacterium nucleatum* infection promotes invasion of oral cancer cells. Sci. Rep..

[106] Kostic A.D., Gevers D., Pedamallu C.S., Michaud M., Duke F., Earl A.M., Ojesina A.I., Jung J., Bass A.J., Tabernero J. (2012). Genomic analysis identifies association of *Fusobacterium* with colorectal carcinoma. Genome Res..

[107] Okuyama K., Yanamoto S. (2023). Oral bacterial contributions to gingival carcinogenesis and progression. Cancer Prev. Res..

[108] Harrandah A.M., Chukkapalli S.S., Bhattacharyya I., Progulske-Fox A., Chan E.K.L. (2020). Fusobacteria modulate oral carcinogenesis and promote cancer progression. J. Oral Microbiol..

[109] Niño J.L.G., Wu H., LaCourse K.D., Kempchinsky A.G., Baryiames A., Barber B., Futran N., Houlton J., Sather C., Sicinska E. (2022). Effect of the intratumoral microbiota on spatial and cellular heterogeneity in cancer. Nature.

[110] Salama A.M., Momeni-Boroujeni A., Vanderbilt C., Ladanyi M., Soslow R. (2022). Molecular landscape of vulvovaginal squamous cell carcinoma: New insights into molecular mechanisms of HPV-associated and HPV-independent squamous cell carcinoma. Mod. Pathol..

[111] Cho Y.A., Kim E.K., Cho B.C., Koh Y.W., Yoon S.O. (2019). Twist and Snail/Slug Expression in Oropharyngeal Squamous Cell Carcinoma in Correlation with Lymph Node Metastasis. Anticancer Res..

[112] Prall F., Ostwald C., Weirich V., Nizze H. (2006). p16(INK4a) promoter methylation and 9p21 allelic loss in colorectal carcinomas: Relation with immunohistochemical p16(INK4a) expression and with tumor budding. Hum. Pathol..

[113] Bertino J.R., Waud W.R., Parker W.B., Lubin M. (2011). Targeting tumors that lack methylthioadenosine phosphorylase (MTAP) activity: Current strategies. Cancer Biol. Ther..

[114] Bataille F., Rogler G., Modes K., Poser I., Schuierer M., Dietmaier W., Ruemmele P., Mühlbauer M., Wallner S., Hellerbrand C. (2005). Strong expression of methylthioadenosine phosphorylase (MTAP) in human colon carcinoma cells is regulated by TCF1/[beta]-catenin. Lab. Investig..

[115] Zhong Y., Lu K., Zhu S., Li W., Sun S. (2018). Characterization of methylthioadenosin phosphorylase (MTAP) expression in colorectal cancer. Artif. Cells Nanomed. Biotechnol..

[116] Amano Y., Matsubara D., Kihara A., Nishino H., Mori Y., Niki T. (2022). Expression and localisation of methylthioadenosine phosphorylase (MTAP) in oral squamous cell carcinoma and their significance in epithelial-to-mesenchymal transition. Pathology.

[117] Yang Z., Zhang C., Qi W., Cui C., Cui Y., Xuan Y. (2018). Tenascin-C as a prognostic determinant of colorectal cancer through induction of epithelial-to-mesenchymal transition and proliferation. Exp. Mol. Pathol..

[118] Gao Y., Nan X., Shi X., Mu X., Liu B., Zhu H., Yao B., Liu X., Yang T., Hu Y. (2019). SREBP1 promotes the invasion of colorectal cancer accompanied upregulation of MMP7 expression and NF-κB pathway activation. BMC Cancer.

[119] Roseweir A.K., Clark J., McSorley S.T., van Wyk H.C., Quinn J.A., Horgan P.G., McMillan D.C., Park J.H., Edwards J. (2019). The association between markers of tumour cell metabolism, the tumour microenvironment and outcomes in patients with colorectal cancer. Int. J. Cancer.

[120] Olianas A., Serrao S., Piras V., Manconi B., Contini C., Iavarone F., Pichiri G., Coni P., Zorcolo L., Orrù G. (2021). Thymosin β4 and β10 are highly expressed at the deep infiltrative margins of colorectal cancer—A mass spectrometry analysis. Eur. Rev. Med. Pharmacol. Sci..

[121] Farah C.S. (2021). Molecular landscape of head and neck cancer and implications for therapy. Ann. Transl. Med..

[122] Bronte G., Silvestris N., Castiglia M., Galvano A., Passiglia F., Sortino G., Cicero G., Rolfo C., Peeters M., Bazan V. (2015). New findings on primary and acquired resistance to anti-EGFR therapy in metastatic colorectal cancer: Do all roads lead to RAS?. Oncotarget.

[123] Komiya Y., Shimomura Y., Higurashi T., Sugi Y., Arimoto J., Umezawa S., Uchiyama S., Matsumoto M., Nakajima A. (2019). Patients with colorectal cancer have identical strains of Fusobacterium nucleatum in their colorectal cancer and oral cavity. Gut.

[124] Kitamoto S., Nagao-Kitamoto H., Jiao Y., Gillilland M.G., Hayashi A., Imai J., Sugihara K., Miyoshi M., Brazil J.C., Kuffa P. (2020). The Intermucosal Connection between the Mouth and Gut in Commensal Pathobiont-Driven Colitis. Cell.

